# Ethical Reasoning During a Pandemic: Results of a Five Country European Study

**DOI:** 10.1080/23294515.2022.2040645

**Published:** 2022-03-09

**Authors:** S. B. Johnson, F. Lucivero, B. M. Zimmermann, E. Stendahl, G. Samuel, A. Phillips, N. Hangel

**Affiliations:** a Ethox Centre and Wellcome Centre for Ethics and Humanities, University of Oxford, Oxford, UK; b Institute for Biomedical Ethics, University of Basel, Basel, Switzerland; c Institute of History and Ethics in Medicine, Technical University of Munich, Munich, Germany; d University College Dublin, Dublin, Ireland; e Department of Global Health and Social Medicine, King’s College London, london, UK; f Centre for Biomedical Ethics and Law, Department of Public Health and Primary Care, KU Leuven, Leuven, Belgium

**Keywords:** Ethics, qualitative research, pandemics, SARS-COV-2, COVID-19, infectious disease, moral judgements

## Abstract

**Introduction:**

There has been no work that identifies the hidden or implicit normative assumptions on which participants base their views during the COVID-19 pandemic, and their reasoning and how they reach moral or ethical judgements. Our analysis focused on participants’ moral values, ethical reasoning and normative positions around the transmission of SARS-CoV-2.

**Methods:**

We analyzed data from 177 semi-structured interviews across five European countries (Germany, Ireland, Italy, Switzerland and the United Kingdom) conducted in April 2020.

**Results:**

Findings are structured in four themes: ethical contention in the context of normative uncertainty; patterns of ethical deliberation when contemplating restrictions and measures to reduce viral transmission; moral judgements regarding “good” and “bad” people; using existing structures of meaning for moral reasoning and ethical judgement.

**Discussion:**

Moral tools are an integral part of people’s reaction to and experience of a pandemic. ‘Moral preparedness’ for the next phases of this pandemic and for future pandemics will require an understanding of the moral values and normative concepts citizens use in their own decision-making. Three important elements of this preparedness are: conceptual clarity over what responsibility or respect mean in practice; better understanding of collective mindsets and how to encourage them; and a situated, rather than universalist, approach to the development of normative standards.

The COVID-19 pandemic has created new ethical contentions. These have emerged not only in professional contexts, such as clinical practice or medical research, but in people’s everyday lives. Should I wear a mask? Should I monitor if others wear a mask? Should I go to work? Should I go on holiday? Should vaccination be mandatory? These overt contentions have been extensively reported in the media, and explored in the scholarly debate, often highlighting their ethical dimensions (Martinelli et al. 2021; Rosen 2021; Sample 2020).

An existing literature on moral pragmatism and implicit normativity (Carter 2018; Cribb 2020; Molewijk et al. 2003; Swierstra and Rip 2007) suggests that where these kinds of overt ethical contentions arise, they are settled not only through explicit ethical deliberation, but also through more implicit normative reasoning (Carter 2018; Cribb 2020; Molewijk et al. 2003; Swierstra and Rip 2007). This means that normative^1^ standards in the pandemic, although sometimes explicit (openly acknowledged and “on the surface”) as ethical debate, can also exist under the surface as “unstated or taken-for-granted assumptions about what is good or bad, right or wrong, required or not required” (Carter 2018). For example, an explicit debate on who should receive COVID-19 vaccines first may carry with it a set of unacknowledged normative assumptions around who we see as most valuable in society (e.g., healthcare workers) or most worthy of protection (e.g., the elderly). Our central claim is that understanding both explicit and implicit normativity with regards to the COVID-19 pandemic in particular, and infectious diseases in general is important. Understanding *explicit* ethical deliberation allows governments and public health authorities to be responsive to public debate. Understanding *implicit* normativity is important so it can be discussed and questioned before becoming invisibly settled in our systems and procedures.

The scholarly literature and debate have so far focused on the overt ethical contentions arising out of the pandemic. Empirical accounts of views on ethical and social issues related to contact tracing apps (Walrave, Waeterloos, and Ponnet 2020; Lucivero et al. 2021), mask-wearing (Betsch et al. 2020; Martinelli et al. 2021), obligations to comply with restrictions (Meier et al. 2020; Neumann-Böhme et al. 2020; Ölcer, Yilmaz-Aslan, and Brzoska 2020; Williams et al. 2020; Wong and Jensen 2020; Zimmermann et al. 2021), and vaccine acceptance and prioritization (Neumann-Böhme et al. 2020; Persad et al. 2021) have been explored. Although valuable, overt ethical contentions are only one part of the ethical discussion. As pointed out by philosopher Alan Cribb, the analysis of less overt norms (or implicit normativity) is “the staple both of philosophical bioethics and the “moral craft” of professional or practical ethics” (Cribb 2020).

To our knowledge, there has been no work that identifies the hidden or implicit normative assumptions on which participants base their views during the COVID 19 pandemic, and their reasoning and how they reach moral and ethical judgements. Here, we refer to morals as norms and values that people use to distinguish between right/wrong, while ethics relates to specific rules, actions, or behaviors about what one “should” do. Morality and ethics are not abstract from the way issues are portrayed and discussed in their own social circles and society more generally. So in trying to understand the moral values people ascribe to in their own decision-making, as well as the unarticulated assumptions that underpin these values, any research/exploration needs to consider not only what these assumptions/values are, but also how they are constructed. The aim of this empirical bioethics study is to gain a deeper understanding of implicit normative positions and morals and ethical reasoning with regards to viral transmission in the COVID-19 pandemic. The rationale for our focus is that viral transmission is the central biological and social “problem” in a pandemic. The premise of one’s deliberation on whether to wear a mask or comply with restrictions is that there is a possibility of disease transmission. Understanding morality and ethics with regards to viral transmission is important in order to clarify the issues at stake in public debates and to make visible patterns of reasoning that influence people’s behaviors and ultimately affect collective action. To this goal, we examined qualitative interview data collected from members of the public in five European countries (Italy, Ireland, Germany, German-speaking Switzerland, and the United Kingdom) during their first lockdowns in April 2020. Our analysis focused on participants’ moral values, ethical reasoning and normative positions around the transmission of SARS-CoV-2.

## Methods

### Research questions

Research questions were developed through an iterative process based on the concepts described in the existing literature on moral pragmatism and implicit normativity (Carter 2018 #4;Cribb 2020 #2;Swierstra and Rip 2007 #3; (Molewijk et al. 2003).

1. Where do participants express ethical uncertainty?
2. What judgements do people express around theirs and others’ behaviors?
3. What ethical arguments or patterns of reasoning are found in the data with regards to viral transmission?
4. What moral values are referred to with regards to viral transmission?

### Data collection

This work is part of the 9-country ‘Solidarity in times of a pandemic: What do people do, and why? A comparative and longitudinal study’ (SolPan) project, which set out to use in-depth interviews and qualitative data analysis methods to investigate and compare the views and practices of people from nine countries in Europe in dealing with the COVID-19 pandemic. The SolPan research commons developed a semi-structured interview guide (SolPan Research Commons 2021b), which included questions on people’s views, experiences, hopes, concerns and expectations about the COVID-19 pandemic, including how they first heard about the pandemic, how their lives had or had not changed as a result, their views on the societal and political response to the pandemic, as well as their hopes for the future. Recruitment took place via e-mail lists, social media, and personal contacts, and interviews were conducted via online platforms or via telephone between 6 April and 6 May 2020. In this paper, data from 177 semi-structured interviews across five of these countries (Germany, Ireland, Italy, Switzerland and the United Kingdom) was analyzed as described below. Table 1 shows self-reported demographic characteristics of participants by country.

### Ethics

Ethical approval for the study was obtained from the University of Vienna Ethics committee Reference Number 00544. The German and Swiss study arm was approved by the Technical University of Munich’s ethics committee (no 208/20 S).

### Data analysis

Across the SolPan Research Commons all interviews were initially coded using a Master Coding Scheme (MCDS) of 129 codes with the assistance of the atlas. ti software (SolPan Research Commons 2021a). The purpose of the initial coding was to be able to sort the interview material into categories or codes for further analysis, and to facilitate working across data-sets (further details forthcoming in: Collaborative Comparisons: Opportunities and challenges in large-scale comparative qualitative research (Hangel and Wegener, Forthcoming)). The codes were used to get access to those parts of the interview that speak to specific research questions. For this study, codes were discussed in a number of online meetings between all authors in the context of broader questions around moral and ethical norms, as well as key literature. From this, the lead author (SJ) developed a series of research questions. Each author then re-analyzed their country data, specifically focusing on the following codes: Moral agency (all codes); Compliance (all codes); Perceiving vulnerabilities (all codes); Ref_disposition twds own country; Ref_other countries; ACT_putting in perspective_general; ACT_putting in perspective persons biography; about the elderly; about the young. Each author produced a report of findings and illustrative quotations. SJ and FL synthesized the findings and produced a first draft manuscript, which underwent several rounds of iteration with all authors.

## Results

Findings are structured in four thematic chapters (Table 2). First, we present participants’ deliberations about ethical contention in the context of normative uncertainty at the beginning of the pandemic due to the inherently novel situation. Second, participants displayed patterns of ethical deliberation when contemplating restrictions and measures to reduce viral transmission. Third, they reported on what they perceived as morally “good” or “bad” behavior. Fourth and finally, participants used existing structures, such as cultural identity, history or normative “facts” of transmission risk or vulnerability, for their ethical reasoning.

### Deliberating and dealing with ethical contention in the context of normative uncertainty

In the early days of the pandemic (spring of 2020), when most countries were facing strict lockdown measures, participants described how they were navigating social contexts without a blueprint of acceptable behavior during a pandemic. These situations were characterized by considered reflections on moral uncertainty and sometimes altercations or negotiations with others about how everyone “should” act.

Participants explained how, as they gained awareness of COVID-19, apparently mundane things such as deciding whether to attend a party or how to behave in a social situation had become a matter of internal debate. This internal debate was often characterized as a balancing act between perceived risks of transmission, previously held socially acceptable norms and personal moral intuitions. For example, one participant described how she balanced perceived risks of contracting the virus on her daily commute with what she felt was socially appropriate (“nice”):

*“In the train it did not seem nice to stay away from people …I did try to avoid [close contact], but if a person sat near me I did not change place, I remained where I was and I tried to stay calm as much as I could, every sneeze seemed to be an impending risk, but I was not so paranoid*.*” (ITFL06)*

The negotiation between different assumptions about ethically acceptable behaviors in the context of the pandemic sometimes materialized in explicit exchanges about what is right or wrong, and altercations among people in public spaces:

“I was in line at the [grocery store], there was a lady who came up and jumped the line, so she stood right next to me. I said, ‘Madam, you are too close. Please step back.’ I told her: ‘Look at the line, it starts there’. I thought I was expressing myself… I mean, I was irritated on my own. Maybe I said it in an unkind way, I don’t know. Anyway, this lady started screaming, saying: ‘You think I don’t know that? Idiot (boor)! How dare you? I can see perfectly well where the queue starts.’ Whereupon I said to myself: ‘Self-control: the lady is out of her mind’. I say: ‘Look, madam, I simply don’t want you to come too close to me. Social distancing is one of the rules, so please comply to it’. Besides, she was an old lady, so I suppose she was more at risk than me.” (ITFL01)

Our participants questioned the moral quality of specific everyday activities and people engaging in those activities. Previously morally neutral activities such as jogging and food shopping, in particular, often acquired a new moral character.

“You see people doing awful things. Joggers and cyclists, oh my God, makes my blood boil. […] Joggers, they keep jogging, they don’t make a move off the path. You have to make a move of the path to avoid them, the two metre rule. They are huffing and puffing, they’re sweating, coughing the whole thing when they go by you…. The jogger thing is more about the fact that they may be infected and not know it and could be giving it to me, that’s where that is coming from and I suppose people not understanding or not realising what actually social distancing means. You know I find going to the supermarket very stressful. It’s fine when you are queuing up, everybody is two metres apart, and then you get inside and people kind are on top of you or reaching across you, or you see people who are clearly over 70 out and about and they shouldn’t be out and about and I kind of worry for them almost” (IESA01)

### Patterns of reasoning when contemplating restrictions and measures to reduce viral transmission

Several participants provided reasons for complying with restrictions and measures to reduce viral transmission. These reasons were diverse and based on different patterns of reasoning and moral principles: many justified their behaviors based on the principle of avoiding harm; some engaged in instrumental reasoning; others were driven by the idea of “doing the right thing” and referred to specific principles such as responsibility and respect for others. Often participants offered a range of reasons.

#### Avoiding harm

Avoiding transmission through compliance with restrictive measures was often justified through a concern for not causing harm either to themselves or others. This was associated with a fear of becoming ill, particularly for people who perceived themselves as particularly vulnerable. “*I am careful because I am a risk patient. I’m not going out of the house, except for taking a little walk*.” (CHO1).

However, many complied not to avoid being infected, but to avoid infecting others and causing harm to them. *“I am not scared for my own health. I’m careful when I go shopping, I put on my plastic gloves and yes, I follow all the rules. But actually more so that I avoid catching it and passing it on to someone else who then has a risk. So in that sense I think we should be careful*.*” (CHBZ19)”*.

This was directed either toward specific people with whom respondents were in a previous relationship (my grandmother, my husband) and who were perceived as more vulnerable, or toward the broader community and non-identified groups of vulnerable people (e.g., older people):

“I want to protect myself and my family, and the wider community, and also I am very aware that I might not be sick, but I could have it on my person and by touching something else and touching somebody else or being you know, close to somebody else I may pass it on to them and therefore the wider community” (IESA01).

Non-maleficence was the most commonly expressed ethical principle across all data, and almost always found alongside other patterns of reasoning.

#### Instrumental reasoning

A second pattern of reasoning was instrumentalist in nature. Namely, compliance to restrictions or advice were aimed at achieving a goal, without a clear reference to the underlining values. This usually took the form of either: we must stop transmission to prevent deaths; or we must stop transmission to get out of lockdown more quickly.

“My immediate family anyway we are taking it very seriously and we do feel like it is impacting our lives and we want other people to take it seriously so we can get out of this quicker” (IESV03).

“But I really want to push this through now, because I really don’t want to start from scratch or ending up in a general curfew. I don’t want that. And I think we are certainly not out of the woods yet.” (CHBZ03)

“I’m [age 50+] Among other things, we don’t just do this because of the old people, but also because of you [younger ones]. Not because you could get so seriously ill, but because you want to go back to your festivals and you want to go out to clubs and dance again. That’s not so important to me anymore, I’m past the age where I need it, but you want to go again. ” (DEBZ09)

Whether the end is to get out of the pandemic, to end up the curfew or going back to festival and clubs, people motivated their compliance to the rules as instrumental to achieving these other goals that they deemed important.

#### Doing the right thing

Many participants referred to the importance of acting responsibly toward the larger society or specific groups, or acting out of respect for other people. This pattern of reasoning shared the feature that people perceived their own daily behavior as part of something with a higher societal meaning. Consequently, even if participants did not agree with the rules, they felt that they must be followed as it was perceived as the “right thing to do.” Instead of focusing on the end goal, in this type of reasoning people referred to principles and norms of behavior that they perceived as right in themselves.

Participants referred to responsibility to not act as a carrier of COVID-19, and to not use or waste healthcare resources, but sometimes they simply referred to the importance of acting responsibly without further articulating what this means. Responsibility was usually directed toward larger society, rather than specific individuals.

“I don’t go out unless I go to the park for a walk or I go to get food, so I try to do my part because I know the healthcare workers are overwhelmed so I take that responsibility and do my part and stay healthy” (IE200428TB02).” I took it very seriously for older people and for risk groups. I saw it more like that, I should respect the curfew or all the other restricting measures. That I should respect them mostly, because it is just so, that I can be a carrier. Not because I am worried about me in any way and I did panic, really. But because of other people, I’d say, to simply take responsibility” (DEBZ08)

Many participants characterized avoiding transmission and adjusting behavior to do so as a matter of manifestation of respect for other people. They rarely articulated what this really meant, indicating it manifest as a general respect for persons, although a few referred to respect for other people’s choices.

“There’s older people that I care about, they come into my shop, so you get to know them all. And they’re standing there and they’re keeping their two meters apart. And then you get other people that come in and stand right on top of them. And they’re not respecting other people’s choices. And I think that needs to change a bit, I think they need to respect each other a bit more” (UKSM05).

Some participants spoke of the importance of self-determination. “We can’t really punish anyone, because at the same time it is a free right to be able to leave your house. It is a free right to be able to do any of the things that we’ve all been doing” (UKSMO5). More commonly, participants referred to those who chose not to follow restrictions as ‘selfish’.

### Moral judgements regarding “good” and “bad” people

Strong judgements were clearly expressed in the numerous quotes where people assessed other people’s behaviors. In condoning their own or others behavior or blaming people’s behavior for virus transmission some respondents effectively divided the world in morally good and bad people. This occurred in patterns with the same groups described as either bad or good.

#### Blaming other people for putting themselves at risk of virus transmission

Participants frequently constructed generational divisions. On the one hand, some blamed the younger generation for not showing any consideration, for “not taking things seriously”.

“It’s a pity to repeatedly see groups of young people. […] We made a bike tour last week and saw how six young people got into a motor boat and drove out to the lake. Like really demonstrating, we won’t be told anything. And I have a hard time with that, because I think the spirit of solidarity would be more important at the moment. If I see something like this, that really makes me angry.” (CHBZ19)

On the other hand, several participants blamed the elderly for exposing themselves to viral transmission at the hairdresser, the supermarket or on public transport.

“[These people] are eighty, ninety years old. I mean, we’ve all seen that people die alone, without even a funeral, intubated. I mean, I can understand a serious reason [to go out], but … obviously the need to have a good haircut is greater than running the risk of dying or ending up in intensive care with a tube! “(ITFL10)

“My children [young adults] and apprentices said, hey, we really stand back and there are really very few people in the trains. But when they are coming home from work in the evening, who is there? The seniors’ groups who went hiking. So for them the issue is: Us, the young, who are basically safe if we got [infected], … we stand back for the elderly, and what do they do? They are happily on the road. That’s polemic, somehow. But I’ve heard that several times now. That’s been difficult for the young.” (CHBZ21)

#### Blaming other countries for virus transmission

Another morally relevant division related to other countries that participants considered as virtuous or blameworthy. Many participants blamed China for the pandemic, for being the source of the virus and for failing to prevent spread outside of China. This was often linked to perceptions about the political environment in China, with the Chinese often described as “secretive”, “unregulated” and “dishonest.”

“I don’t want to contradict that and saying I believe everything I saw, but just reading about what the cases per day, how it was rising in China and then realising that they are not recording it properly and even like now they still haven’t probably recorded properly. I think that was quite shocking and it annoyed me a lot when the Chinese Government didn’t seem to comply with a lot of the regulations and advice on measuring the amount of people that have had corona. I think that China didn’t comply with a lot of those regulations, but that’s just what I’ve read. So, a lot of China’s reaction and the way they, because it’s China a lot of information is censored so there is never a full transparent view of what actually is happening” (IEES02)

Participants often also blamed other countries for virus spread in their own countries. For example, German participants sometimes blamed Austria (for leaving ski resorts open), some Swiss participants blamed Italy (for not being careful enough).

### Using existing structures of meaning for moral reasoning and ethical judgment

In the context of new normative uncertainty participants drew on a variety of resources to make ethical judgements. This included “facts” about risk and vulnerability to severe disease, their cultural identities, their life experience and historical crises.

#### Normative “facts” about risk of transmission

Judgements about ethical practice or behavior were heavily tied to both the perceived value of an activity, and the perceived risk of transmission associated with the activity. Perceptions of transmission risk varied by context, and in turn the normative connotations associated with specific activities varied by context also. Which activities were deemed to be particularly high risk, and therefore morally bad to engage in, varied by country. Italian participants, for example, tended to moralize around behaviors with regards to people moving around. It is important to note that the restrictions in Italy focused heavily on limiting movement during April 2020.

“Some people have locked themselves in the house like us or stayed at home, unless they went shopping or to the pharmacy, while other people took a little walk, went to see their the girlfriend in the near village. […] If everybody did their part really, they would have been home for two months […] I mean my neighbour definitely went to his girlfriend. We didn’t see him for two days. Where did he go? He must have gone [laughs]. But I understand it, I can also understand it. But it’s not fair, that’s it. Because you have to think even a little bit about the others, the others who are inside the house. That is, if I am at home, I think you should be too. I am not going to report or shoot someone because he went to his girlfriend on Easter day. But come on… There are those who have done it and those who have not.” (ITIG13)

We did not see this focus on morally bad behavior with regards to “unnecessary” movement in the other countries investigated. Instead, participants in the UK, German and Swiss data, where restrictions focused on limiting the number of people mixing socially tended to moralize around people gathering in large groups.

“So sometimes I get totally annoyed when I see people sitting around in the park. More than five people or not far apart. It really annoys me. I just don’t think that’s okay. They just don’t get it. But then you really do see people walking with a distance between them. They go jogging or walking. And that I find good.” (CHBZ04)

Thus, participants judged the ethical (il)legitimacy of people’s behavior in perceived objective facts about what behavior causes high risk of viral transmission, even though those “facts” were in fact heavily shaped by the context and country-specific policy strategies.

#### Normative “facts” about vulnerability

People identified as particularly “vulnerable” during the pandemic included those perceived to be clinically more susceptible to severe illness, usually the elderly and people with asthma or cancer, as-well-as those perceived to be socially vulnerable. The latter included people working in exposed and risky jobs, people living alone, young and/or single parents and children (who may be missing school and social development).

“I worry about my grandmother, she lives alone. And she does has someone who goes grocery shopping for her, but for her it is much worse than for me. Because I live with my family, but she lives completely alone, more or less.” (CH01)

No participants identified people with obesity, smokers or black and ethnic minorities as vulnerable groups (with the exception of one UK participant who identified themselves as high risk due to their ethnicity). As such, “facts” around who was vulnerable to virus transmission did not align with biological realities.

#### Cultural identities

Across all data sets some of the particpants drew on their cultural identities to assess what was permissiable, right/wrong or ethically (il)legitimate responses to the pandemic and restrictions. Where this happended, they often drew on stereotypes about their own countries and others.

Some UK participants spoke exceptionally about the “UK culture” and how the political/social climate was different in the UK to other countries. In particular, participants deemed the protection of civil liberties ethically more important in the UK than elsewhere.

“Some countries like Spain and Italy you’re reading things in the press that they’re getting fined 10 000 Euros if they’re caught out of the house when they shouldn’t be. We’re given £60 fines after the fourth time of telling them to move on. So I think, I don’t think we should be as harsh as the other countries because I think that’s what makes the UK a great place to live.” (UKSH03)

Swiss participants tended to refer to economic security and how this conferred advantage and a responsibility to accept restrictions (because they know they get help from the state).

“People accept [the restrictions] because we are very well secured here. We have our rights, we have very good help for the individual and small businesses. […] Certainly the costs are very high, which we pay here. And I think that we also have the right. Well, not necessarily, but I do think that we are now benefiting from the fact that we have made provisions, in the financing of Switzerland, that they can now give it back to us.” (CHBZ20, 21:15)

Irish participants tended to refer to a history of oppression, and how this shaped attitudes to compliance.

“I think culturally as a nation you know you can put it back to sort of, without getting too nationalistic, 800 years of oppression and a natural inclination to kind of nod and say oh yeah there the rules, that’s great and then just do what we want to on the down low and on the QT. And I think that’s culturally embedded in our psyche to be quite honest with you” (IE200503SA01).

Italians were perceived to being “sly” and rule breakers by some Italian participants.

“So obviously they have closed everything and yet you can still see the sly one every now and then, there is the sly one, typical Italian sly one, who circumvents the ban and goes there, then they regularly get the fine obviously because they are checked and maybe they go jogging on the meadows, where you can’t. If you can’t go jogging you don’t jog” (IT200420LM03).

In addition to a general rule oriented German stereotype, the discussion about how to apply rules and recommendations became more nuanced (see also Zimmermann et al. 2021).

“In Germany, as far as I’ve seen it here in [city], the people are quite disciplined and keep their distance. I have the feeling that people tend to be nicer than they usually are, a little friendlier, a little more accommodating. So I have the feeling that the crisis is leading to a certain extent that we are a little more careful about each other at the moment. […] But right now I can see that people are trying hard, that they are trying to be patient, that they are sticking to that by and large. Of course there are always outliers, but the majority of the people here actually agree with the measures, see the need for them and also stick to the recommendations.” (DEBZ10)

#### Life experience and historical crises

Participants drew on a number of preexisting experiences to make sense of the pandemic and often to inform ethical judgements about acceptable behavior. For example, healthcare workers and people in the food industry tended to be very supportive of strict infection control, drawing on their training and experience in this area. Others drew on real or imagined experiences of previous disease outbreaks or the Second World War to determine the required “attitude” to navigate the pandemic.

“But I think it’ll be a bit of World War Two nostalgic feeling. Rolling your sleeves up and come on we can do better than this. We can get out of this. And I think that’s what we need as a nation and I think we can supply the nation under the right leadership.” (UKSH03)

“The first thought it was like any other disease that was reported in the news over the last 10 years or 15 years like Ebola or like the bird flu, SARS, MERS because I have worked in Dubai for 10 years and we have back there was news on MERS, the Middle East Respiratory Syndrome, so yes there are concerns, but it is not to the extent where you are not allowed to go out etc. Life was still normal even with SARS or MERS at the time. This is definitely a gamechanger, a different type of impact this disease has to our normal life.” (IE200501ES05)

## Discussion

Our participants reasoned through their experiences, actions and the action of others based on their values, their worries about others, the role that they see themselves playing, the meaning and importance they gave to different normative concepts, such as responsibility. They displayed recognizable patterns of moral judgements and ethical reasoning. Moral tools, therefore, were an integral part of people’s reaction to and experience of the pandemic, not a corollary. Our findings are consistent with a large literature in moral psychology that demonstrates that moral convictions guide many of our thoughts, behaviors, and social interactions (Feinberg et al. 2019), and that moralizing moves people to engage in behaviors in line with such moralization (Skitka 2010). In other words, if I think an action is ‘right’ or ‘good’, I am more likely to do it. The understanding of the moral component in people’s behaviors and reasoning has obvious implications for encouraging compliance with public health interventions during a pandemic. If we understand people’s normative standards, we can use this to encourage them to comply for the benefit of themselves and others. A better understanding of people’s moral convictions and patterns of reasoning may, therefore, have an important part to play in combatting the COVID-19 and future pandemics.

While moral tools were clearly essential, an understanding of the moral values and ethical reasoning citizens use in their own decision-making about the pandemic are lacking and have not been previously explored. In this study participants moral values and ethical reasonings were often heterogeneous, inconsistent (giving both instrumental and rule governed justifications for action), assumed rather than critically evaluated, and showed troubling patterns of replicating existing stigmas and preconceived ideas about specific groups (e.g., the young). It is our contention that participants were, therefore, “morally unprepared” for the pandemic. “Pandemic preparedness,” including “ethical preparedness” have become an important element of public health discussion on how to anticipatory develop a framework and infrastructure to guide public authorities’ responses to pandemic crisis (Fenton, Chillag, and Michael 2015; Thompson et al. 2006). Ethical preparedness has been described as a way to “illuminate the values at stake in [public health] decisions and provide the moral language to describe and resolve situations in which values conflict” (Leslie Meltzer Henry 2019). It is our contention that while important, what is lacking here, is another form of “moral preparedness,” which consists of an understanding of the moral values and normative concepts citizens (not policy makers) use in their own decision-making about the pandemic. A deeper understanding of citizens’ moral values and ethical reasoning is important because it can help guide citizens as well as decision makers in their response to the pandemic. It can also work to enhance public dialogue through developing a shared moral language, and to set new normative standards. All of this may reduce moral distress, stream line decision-making and improve outcomes (human flourishing). In the below, we discuss our data by reflecting on what is needed in order to develop moral preparedness going forward.

### The need for empirical and normative work around responsibilities, respect and moral obligations

Our findings were consistent with literature that shows that in practice, people do not reason in the straight lines of ethical theory. For example, in their work Kahane and colleagues found that even when people endorse consequentialist principles in the abstract, their actual moral judgments may still be guided by deontological considerations relating to rights, duties, or degrees of personal relationship (Kahane et al. 2018; Tanner, Medin, and Iliev 2008). In our data, we saw a strong focus on avoiding harms, often accompanied by consideration of principles and values such as respect and responsibility. This indicates that a broad range of moral thought and judgment, play a role in ethical judgment. While the consequences of transmission of SARS-COV-2 may be relatively easy to identify and agree upon (illness, death, economic crisis), and were consistently represented in the data, moral principles were understood in different ways and poorly articulated. There was no conceptual clarity over what responsibility or respect meant in practice: who are we responsible to; how responsibility and respect manifest (what obligations in practice does this confers)? In this study, participants were not asked to elaborate on concepts related to the research question (to avoid socially desirable answers regarding ethics). Further empirical work, which investigates how these concepts are understood by members of the public, and what role they play in ethical reasoning, is needed. This will support the development of a “shared moral language,” meaning research and policy may take account of and respond to public values more easily. Further normative work addressing the relevant principles, and how this relates to the proper policy response is also required.

### Vulnerability and risk as socially constructed

Participants shared a fairly uniform view of the potential consequences and harms of SARS-COV-2 transmission. Namely illness for themselves or others, particularly “vulnerable” others. Yet, there was evidence that implicit normative judgements were embedded in how people constructed vulnerability. No participants identified people with obesity, smokers or black and ethnic minorities as particularly vulnerable groups. Given that it has been empirically demonstrated that these groups are more vulnerable to severe disease (Kwok et al. 2020; Sze et al. 2020; VAN Zyl-Smit, Richards, and Leone 2020), and are already stigmatized this may represent a troubling finding about existing normative commitments with regards to who matters and who is worthy of protection. Being morally prepared in this context would require us to take into account how people’s understanding of potential harms and vulnerabilities in disease transmission, is socially constructed, imbued with values and not just a matter of scientific fact. In our data perceptions about vulnerability often reflected existing power imbalances and inequities. Similarly, we saw how perceptions of “high risk behavior” were influenced by the local policy context. This understanding can be used to anticipatory develop strategies to reduce inequities and negative impacts of policy discourses.

### Implications of a blaming dynamic and culture of responsibilisation

In our study, a “blaming dynamic” played a role in the attribution of moral status and judgment to specific groups. This was often based on preexisting assumptions and beliefs, regarding certain communities (e.g., the young). A culture of responsibilisation of individuals and specific groups is dangerous in these contexts. First, because it distributes responsibilities among social actors in way that can first of all impact the most vulnerable and stigmatized communities. Second because it may divert attention away from those who have the power to affect change (governments etc.). Third, because it breaches a culture of cohesion and solidarity, at a time when that is of crucial import (Nuffield Council Of Bioethics 2020). Evidence suggests that social cohesion and community resilience make communities better able to cope with crisis situations (Lalot et al. 2021) and are important resources in the recovery after any disaster(Jewett et al. 2021). Better understanding collective mindsets and how to encourage them will be important going forward.

### Moral preparedness is not universal

In highlighting the role of cultural identities and the self-positioning of our participants with respect to “others”, our study suggests that an approach to moral preparedness is not based on universal ethical principles but on thick descriptions and deep interpretations of values, identities and rules circulating among different social groups and cultures. Pragmatist philosophy literature, especially in its formulation by John Dewey, has highlighted that moral reasoning is situated in cultural contexts and can be understood only by engaging with concepts and values that are specific to a certain culture (Dewey and Boydston 1981). The centrality of this approach to successful pandemic response is supported by social science from previous epidemics and disease outbreaks (Bavel et al. 2020). A situated, rather than universalist, approach to moral preparedness means to think about how the concepts and values that are specific to a certain culture may have implications on behaviors, adherence, and resistance to policy control measures. For example, the centrality of the value of community and family relationships in some cultures will make some of the restrictions regarding social distancing or social bubbles much more difficult to understand and adhere to by citizens. It is important to consider moral situatedness not only across countries but also within the same country. Both policy measures and collective responses are likely to be influenced by meanings and cultural identities that circulate within different for different social groups.

### Limitations

Our sample was skewed toward white, educated middle-aged adults (see Table 1 reporting demographic data). This means important perspectives are missing from the data. Results may have differed significantly in other socio-economic and racial groups. Both a strength and weakness of the study is that it captures a particular moment in time – the first lockdown in each country. It is likely that over time normative uncertainty and positions has changed. For example, new issues may have emerged. It is also likely that people’s moral attitudes and reasoning have developed over time. Therefore, this study can be considered relevant to the early phases of a pandemic and provides an example of how ethical reasoning changes in immediate moments of public health crises. It’s also possible participants gave socially desirable answers as few participants admitted to breaking the rules. Although, we specifically refrained from asking participants about their moral attitudes to avoid this.

## Conclusion

Moral tools are an integral part of people’s reaction to and experience of a pandemic. Our study shows that in the early phases of the COVID-19 pandemic participants across five European countries were morally and ethically unprepared. “Moral preparedness” for the next phases of this pandemic, and for future disease outbreaks, epidemics and pandemics will require an understanding of the moral values and normative concepts citizens use in their own decision-making. We have identified three important aspects that need to be further articulated in order to develop such preparedness: conceptual clarity over what responsibility or respect mean in practice; better understanding of collective mindsets and how to encourage them; and the development of a situated, rather than universalist approach to the development of normative standards.

## Figures and Tables

**Table 1:** Self-reported demographic characteristics of participants by country (T1).

Category	UK (*n* = 35)		DE (*n* = 46)		IT (*n* = 33)		IE (*n* = 32)		CH (*n* = 31)	
**Age**										
18–30	6	17%	9	20%	3	9%	5	16%	8	26%
31–45	11	31%	19	41%	15	45%	13	40%	6	19%
46–60	11	31%	5	11%	8	24%	8	25%	7	23%
61–70	5	14%	8	17%	3	9%	2	6%	5	16%
70+	2	6%	5	11%	4	12%	4	12%	5	16%
**Gender**										
Female	20	57%	24	53%	22	67%	20	62%	16	52%
Male	14	40%	22	47%	11	33%	12	37%	15	48%
Other	1	3%	0	0%	0	0%	0	0%	0	0%
**Household**										
Single	4	11%	13	28%	7	21%	9	28%	8	26%
Couple	13	37%	16	35%	8	24%	11	34%	10	32%
Living with child/children under 12	8	23%	8	17%	6	18%	5	16%	3	10%
Living with child/children 12+	4	11%	4	9%	5	15%	6	19%	5	16%
other	6	17%	5	11%	7	21%	1	3%	5	16%
**Rural/urban**										
Big town (e.g., capital, +500k)	5	14%	22	48%	14	42%	17	53%	10	32%
Medium/small town	18	51%	12	26%	11	33%	10	31%	6	19%
Rural (e.g., village)	12	34%	12	26%	8	24%	5	16%	15	48%
**Employment status**										
Employed (long-term contract)	17	49%	21	52%	10	30%	16	50%	13	42%
Self-employed	5	14%	4	9%	9	27%	4	12%	3	10%
Employed (short-term/precarious contract)	2	6%	3	0%	3	9%	2	6%	6	19%
Unemployed	4	11%	4	9%	2	6%	2	6%	1	3%
Retired	5	14%	10	21%	3	9%	4	12%	7	23%
other	2	6%	4	9%	6	18%	4	12%	1	3%
**Education level**										
Less than 10 years	2	6%	2	4%	2	6%	2	6%	10	32%
10–14 years (e.g., highschool diploma)	10	29%	16	35%	17	52%	3	9%	3	10%
Higher education	23	66%	28	61%	14	42%	27	84%	18	58%
**Household net income** (prior to Corona), net income:										
Up to 1,400€(1200GBP/4000CHF)/month	5	14%	5	11%	5	15%	3	9%	6	19%
1,401(1201)–3,000€(2600GBP/4001-7000CHF)/month	5	14%	14	30%	22	67%	9	28%	9	29%
More than 3,000€(2600GBP/7000CHF)/month	25	71%	27	59%	6	18%	20	62%	16	52%

**Table 2:** themes and sub-themes.

Theme 1	Theme 2	Theme 3	Theme 4
**Deliberating and dealing with ethical contention in the context of normative uncertainty**	**Patterns of reasoning when contemplating restrictions and measures to reduce viral transmission**	**Moral judgements regarding “good” and “bad” people**	**Using existing structures of meaning for moral reasoning and ethical judgment**
	Avoiding harm Instrumental reasoning Doing the right thing	Blaming other people for putting themselves at risk of virus transmission Blaming other countries for virus transmission	Normative “facts” about risk of transmission Normative “facts” about vulnerability Cultural identities Life experience and historical crises

## Data Availability

Due to the nature of this research, participants of this study did not agree for their data to be shared publicly, so supporting data is not available.

## Note

1 Here we take *normative* in opposition to *descriptive*: while descriptive claims depict a state of affairs, normative claims make claims on us, they command, oblige, recommend, or guide CARTER, S. M. 2018. Valuing Healthcare Improvement: Implicit Norms, Explicit Normativity, and Human Agency. *Health Care Analysis*, 26, 189-205.
